# Pentavalent vaccination in Kenya: coverage and geographical accessibility to health facilities using data from a community demographic and health surveillance system in Kilifi County

**DOI:** 10.1186/s12889-022-12570-w

**Published:** 2022-04-25

**Authors:** Morris Ogero, James Orwa, Rachael Odhiambo, Felix Agoi, Adelaide Lusambili, Jerim Obure, Marleen Temmerman, Stanley Luchters, Anthony Ngugi

**Affiliations:** 1 Department of Population Health, Aga Khan University, Nairobi, Kenya; 2 School of Mathematics, University of Nairobi, Nairobi, Kenya; 3 Health Services Unit, KEMRI-Wellcome Trust Research Programme, Nairobi, Kenya; 4 Institute for Human Development, Aga Khan University, Nairobi, Kenya; 5 Centre of Excellence for Women and Child Health, Aga Khan University, Nairobi, Kenya; 6 Department of Obstetrics and Gynaecology, Aga Khan University, Nairobi, Kenya; 7 International Centre for Reproductive Health, Department of Public Health and Primary Care, Ghent University, Ghent, Belgium; 8 Department of Epidemiology and Preventive Medicine, Monash University, Melbourne, Australia; 9 Burnet Institute, Melbourne, Australia

**Keywords:** Pentavalent, Geographical accessibility, Travel time

## Abstract

**Background:**

There is substantial evidence that immunization is one of the most significant and cost-effective pillars of preventive and promotive health interventions. Effective childhood immunization coverage is thus essential in stemming persistent childhood illnesses. The third dose of pentavalent vaccine for children is an important indicator for assessing performance of the immunisation programme because it mirrors the completeness of a child’s immunisation schedule. Spatial access to an immunizing health facility, especially in sub-Sahara African (SSA) countries, is a significant determinant of Pentavalent 3 vaccination coverage, as the vaccine is mainly administered during routine immunisation schedules at health facilities. Rural areas and densely populated informal settlements are most affected by poor access to healthcare services. We therefore sought to determine vaccination coverage of Pentavalent 3, estimate the travel time to health facilities offering immunisation services, and explore its effect on immunisation coverage in one of the predominantly rural counties on the coast of Kenya.

**Methods:**

We used longitudinal survey data from the health demographic surveillance system implemented in Kaloleni and Rabai Sub-counties in Kenya. To compute the geographical accessibility, we used coordinates of health facilities offering immunisation services, information on land cover, digital elevation models, and road networks of the study area. We then fitted a hierarchical Bayesian multivariable model to explore the effect of travel time on pentavalent vaccine coverage adjusting for confounding factors identified a *priori*.

**Results:**

Overall coverage of pentavalent vaccine was at 77.3%. The median travel time to a health facility was 41 min (IQR = 18–65) and a total of 1266 (28.5%) children lived more than one-hour of travel-time to a health facility. Geographical access to health facilities significantly affected pentavalent vaccination coverage, with travel times of more than one hour being significantly associated with reduced odds of vaccination (AOR = 0.84 (95% CI 0.74 – 0.94).

**Conclusion:**

Increased travel time significantly affects immunization in this rural community. Improving road networks, establishing new health centres and/or stepping up health outreach activities that include vaccinations in hard-to-reach areas within the county could improve immunisation coverage. These data may be useful in guiding the local department of health on appropriate location of planned immunization centres.

## Background

The third Sustainable Development Goals (SDG) targets reducing childhood mortality from preventable deaths is ensuring universal vaccination coverage [1]. Estimates put lives saved through immunization at 2–3 million per year [2], which is substantial evidence that immunization is one of the most significant and yet cost-effective pillars of preventive and promotive health interventions [3]. The establishment of the World Health Organization’s Expanded Program of Immunization (EPI) resulted in the introduction of more vaccines and better global coverage. The coverage of initial core vaccines (Bacille Calmette -Guerin (BCG), Diphtheria-Tetanus-Pertussis (DTP), Polio, and measles vaccine) increased from 5% in 1974 to over 86% in 2018 [4, 5]. Despite impressive global statistics, there are substantial inter- and intra-country heterogeneities of vaccine coverage resulting in approximately 19.4 million unimmunised children in 2018. The majority of these children are from sub-Saharan African (SSA) countries [4, 6], where the mortality rate from vaccine-preventable diseases for the under-fives remains among the highest in the world [7].

The third dose of pentavalent vaccine for children is an important indicator for assessing performance of the immunisation programme because it mirrors the completeness of a child’s immunisation schedule [5]. For this reason, the Global Vaccine Action Plan (GVAP) set a dual target for pentavalent vaccination at 90% in national coverage and 80% for other administrative units by year 2020 [8]. According to the Global Alliance for Vaccines and Immunizations (GAVI), Kenya national estimates of pentavalent coverage were 81% in 2018 [9]. However, there is potential masking of spatial heterogenicities, especially in rural areas or areas of low coverage, as a result of averaging across regions. This might allow pockets of preventable infectious diseases to persist [10], which could act as foci for potential future outbreaks.

Geographical access to a health facility offering immunization services is a significant determinant of pentavalent vaccination coverage, as the vaccine is mainly administered during routine immunisation schedules at health facilities [11–13]. Studies have shown that rural areas [14, 15] are most affected by poor access to healthcare services. Although factors that influence access to immunisation services have been studied extensively in a broader sense [12, 16–20], the local context within communities, which to a larger extent determines how these factors interact, has not been explored. Furthermore, the role of geographical access to primary health services is poorly described in Kenya. In this study, we sought to determine vaccination coverage of pentavalent vaccine, determine geographical accessibility to health facilities offering immunisation services, and explore its effect on immunisation coverage in one of the predominantly rural counties (Kilifi) on the coast of Kenya.

## Methods

### Study area

We utilized longitudinal survey data from the Kaloleni-Rabai Community Health Demographic Surveillance System (KRHDSS) in the coast of Kenya. This system is nested on the local community health infrastructure and that regularly captures demographic and health information and vital status and migration in a local community. It tracks a cohort of more than 92,000 population in over 18,000 households and covers 113 villages in this area. Households of interest in our study were those with children aged between 14 weeks and 11 months. The 113 villages are distributed among 10 Community Health Units (CHU), which are the lowest-level tier in the Kenyan health system structure interfacing the health system on one hand and the households on the other. Three CHUs (Buni, Vishakani, Mwele-Kisurutini) were considered peri-urban as they were adjacent to urban areas or encompassed parts of local rural towns within the sub-counties of interest. The cohort has been followed up semi-annually since 2017 and by 2019, six rounds of data collection had been completed. Longitudinally linked individual level information (using unique identification numbers) was collected during each round. We accessed the data in December 2019 and identified 4,442 eligible children aged 14 weeks to 11 months from the cohort for the purposes of this study. This age-bracket represents the optimal times to assess coverage of pentavalent vaccination as recommended within the Kenya’s Ministry of Health community health data collection guidelines [21], as it is during this period that the three doses of pentavalent vaccine are considered complete.

New individuals can enter this cohort by either birth or in-migration, while cohort members can exit by either out-migration or death. A detailed profile of this cohort has been presented elsewhere [22].

### Data collection

For each round of data collection, a trained community health volunteer (CHV) visited the longitudinally tracked households and interviewed the mother or caretaker of the child who provided the following data: vaccination data (based on child’s vaccination card or on maternal recall if card is unavailable), demographic information, reproductive, maternal and child-health data, child orphan status, school attendance among children, social determinants of disease (e.g. insecticide-treated bed-net use, Water, Sanitation and Hygiene (WaSH) practises, access to HIV testing etc.), child nutritional data (MUAC measurements),vital events (births, migration, and deaths) and pentavalent immunization data for all children 14 weeks – 11 months of age. Global positioning system (GPS) coordinates of the households were also collected. A preconfigured open data kit (ODK) installed in electronic tablets was used for data collection, and upon completion of the interview, data were reviewed for completeness and synced to a central server. Further data screening was performed by a data manager for any errors (omissions and inconsistencies) and the feedback sent to CHV for verification. The whole process of data collection was supervised and coordinated by KRHDSS field officers and the local public health personnel.

#### Estimation of geographical accessibility

**Fig. 1:**
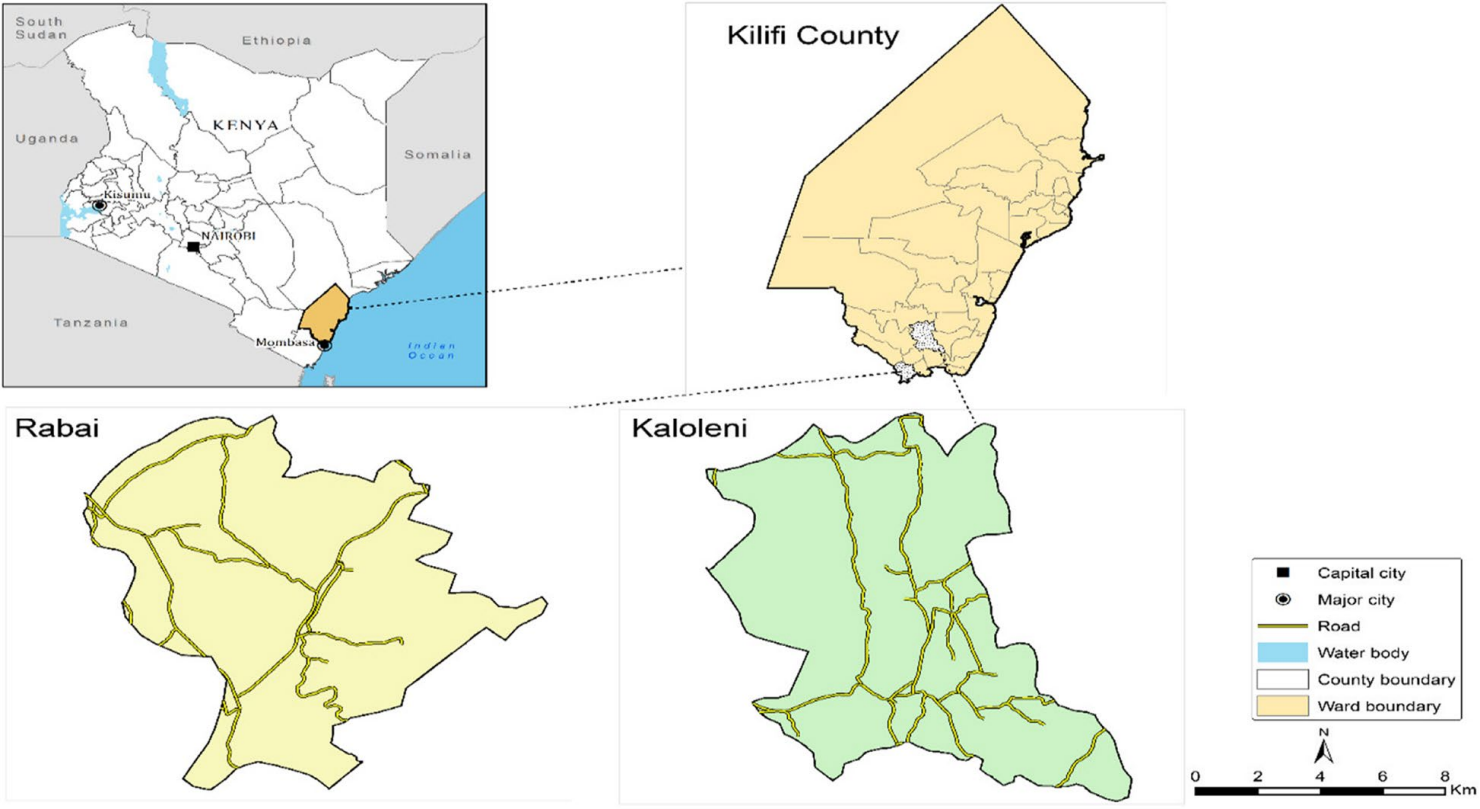
Map of Kaloleni-Rabai Subcounties where the Community Health Demographic Surveillance System is implemented. Source of map: *generated by the author using open-source software QGIS v3.12*

We assembled information on coordinates of health facilities, land cover, digital elevation model, road network, and barriers within Kaloleni and Rabai sub-counties in Kilifi County (Fig. 1) to compute travel time which is a marker of geographical accessibilities of health facilities offering immunization services.

#### Health facilities

**Fig. 2:**
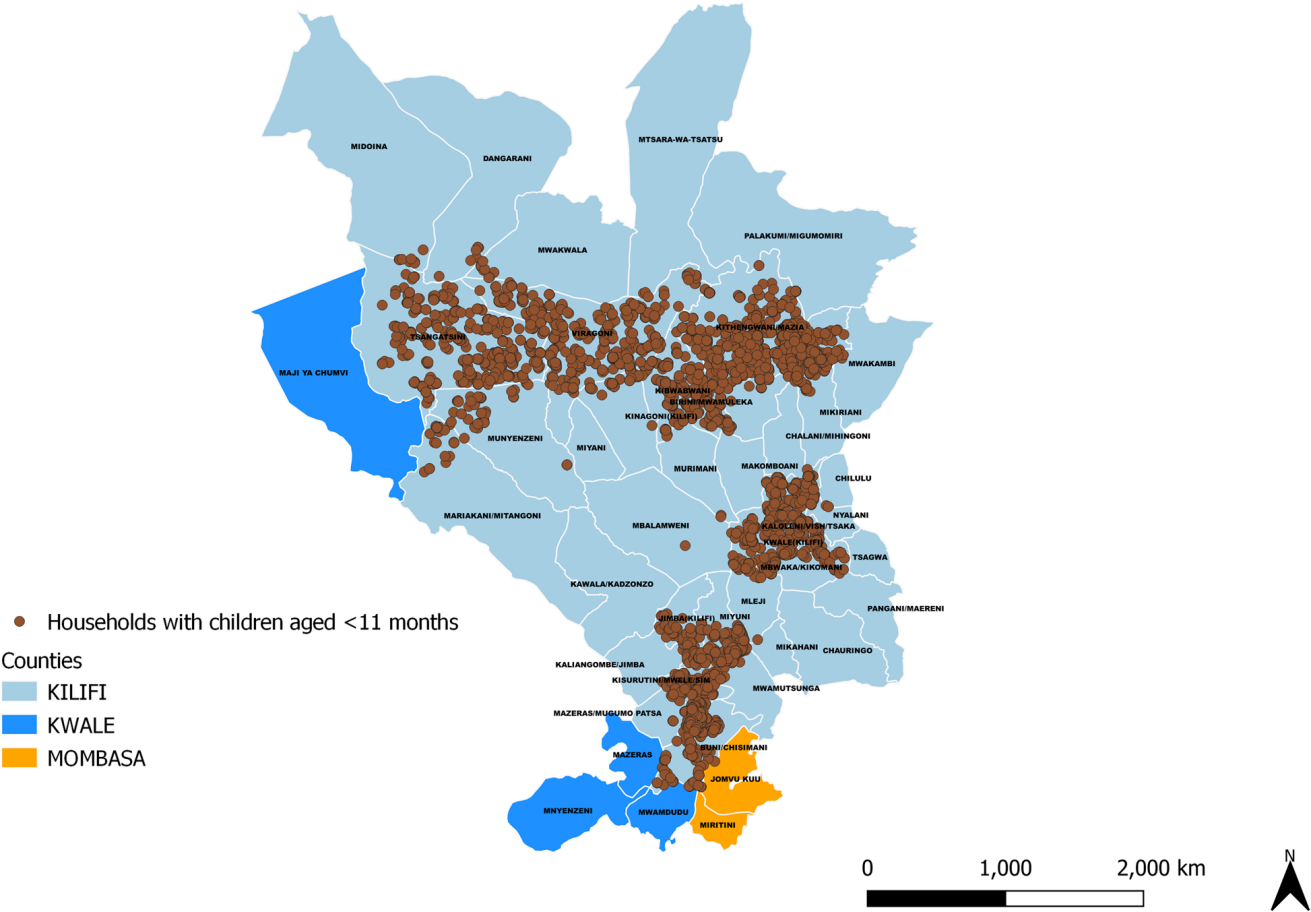
Map showing the distribution of households with children aged < 11 months in the study area*.* Source of map: *generated by the author using open-source software QGIS v3.12*

We obtained a list of all facilities that offer immunization services within the study area from the Kenya master health facility list [23] and the Kenya health information system [24]. We merged facilities from these two sources, eliminated duplicates and obtained their GPS coordinates, which we validated against the recently geocoded master database of all health facilities in sub-Saharan Africa [25]. We also included health facilities from the sub-counties neighbouring the study area, with the assumption that the nearest health facility might be in a neighbouring sub-county especially for household along the borders of the study area as shown in Fig. 2. Further, we ensured that the resultant heath facilities were within the settlement and not on waterbodies by checking their coordinates using Google Earth.

#### Road Network

Data for road networks were assembled from OpenStreetMaps (OSM) and Google Map Maker (GMM). Duplicates and short sections of roads disconnected from the main network were removed. As done elsewhere [26, 27], we classified roads into 4 categories: primary (class A & B) roads that mainly connect international borders, secondary (class C & D) roads that feed into primary roads or connected to major towns, county (class E) roads that feed into secondary roads and connect smaller towns or market centers, and rural (class U) roads that connect rural areas. These roads were assigned different speeds depending on the probable mode of transport as follows: primary and secondary roads whose modes of transport were vehicular were assigned speeds of 80 km/h and 50 km/h, respectively. County roads with bicycling as a mode of transport were assigned 11 km/h, while rural roads were assigned 5 km/h based on similar studies in Kenya [27, 28].

#### Digital elevation model & land cover

We obtained data for the land cover and digital elevation model (DEM) at a spatial resolution of 30 m from the Regional Centre for Mapping of Resources for Development (RCMRD) [29]. This is the centre responsible for disseminating open geospatial datasets for Eastern and Southern Africa. Land cover for the study area consisted of 9 categories, which we assigned walking speed based on previous studies [27, 28, 30]; tree cover (4 km/h), shrub cover (5 km/h), grassland (5 km/h), cropland (2 km/h), aquatic vegetation (0.01 km/h), sparse vegetation (2 km/h), bare areas (5 km/h), built-up areas (5 km/h), and open water (0.01 km/h). Walking and bicycling speeds were further adjusted accordingly based on the topography derived from the DEM. This correction used Tobler's equation [31] that linked walking and bicycling speeds with the slope of the terrain.

$$W=6\ast\exp\left(-3.5abs\left[\mathit{tan}\left(\frac S{57.296}\right)+0.05\right]\right),where\;W,\;is\;the\;speed\;calculated\;and\;S\;is\;the\;slope\;in\;degrees$$

Land covers and the DEM showing different elevations of the study area are provided in supplementary file 1.

#### Estimation of travel time

Methods for estimating geographical accessibility have been developed over time, namely, the travel time model [26], network analysis [32], and gravity model [33]. In this study, we used the travel time model because it has been recommended by the WHO as a suitable method of modeling healthcare accessibility [34] and because it takes into consideration other key aspects of accessing care, such as terrain and land cover surfaces [35].

We used AccessMod (version 5) [36] to model geographical accessibility. The software uses the Manhattan distance method to cumulatively determine the time needed to cross contiguous cells using the least cost path from settlement to immunizing health facilities. Therefore, to estimate travel time, we first generated a travel impedance raster surface by merging land cover, elevation, and road network. To each contiguous cell of the resultant raster layer, we assigned travel speeds accordingly as described earlier. Lastly, we combined the location of the immunizing health facilities to the rasterized layer and estimated the time in minutes needed to travel to the nearest facility at 30 m spatial resolution. For further analyses, we extracted the travel time for each household’s geographical coordinates from the generated raster file. The obtained travel time was then assigned to children within a given household. Maps of travel time to the nearest immunizing health facility and the average time per household were plotted in QGIS (version 3.12).

### Statistical Analyses

In our analyses, we included other factors likely to influence the association between travel time and uptake of pentavalent vaccination, either as confounders or effect modifiers. These included i) location of the household of interest (rural or peri-urban), surrogates of contact with health facilities for services other than for pentavalent immunization, and iii) individual characteristics (e.g. whether the index child was an orphan based on our previous findings from the area [37] and uptake of health behaviors such as use of insecticide treated bed nets, and positive WaSH practices). We used a Bayesian hierarchical logistic regression model to explore the effect of geographical accessibility on pentavalent coverage on the population of 4,442 children aged 14 weeks to 11 months in the cohort. Community Health Units (CHUs) and round of data collection were used as random effects. To stabilize computations, we used weakly informative priors that also served to bind the estimates within the acceptable ranges [38]. We specified four chains each with 5000 iterations, half of which were used to warm the sample and were discarded before estimations were made. The convergence of the model was determined by examining trace plots of the model. We adjusted for confounding due to sociodemographic and other factors described above. In keeping with previous studies investigating the effect of travel time [28], we grouped travel time into two groups: less than 1-h and more than 1-h travel to a health facility. To compare differences between two groups, we used an *independent t-test* statistical technique, and the results were interpreted using a *p-value* at the significance level of α = 0.05. The results from the multivariable model were reported as odds ratios (ORs) and 95% credible intervals. Significance of odds ratios was assumed if the 95% credible intervals excluded one. All analyses were performed using R Version 3.4.3.

## Results

### Demographic characteristics of the sample

We found that majority of the children from our sample were female (2,261, 51%). The median age was 7.4 (IQR 5.5–9.1) months. The median number of children per CHU was 303 (IQR = 181 – 404). Demographic characteristics were not significantly different between vaccinated and unvaccinated children, as shown in Table 1.

**Table 1:** Characteristics of the children 14 weeks to 11 months eligible for pentavalent vaccination

**Characteristic**	**Pentavalent vaccinated (*n* = 3435)**	**Pentavalent not vaccinated (*n* = 1007)**	**Overall (*n* = 4442)**
Gender (Female)	1743 (50.7%)	518 (51.4%)	2,261 (51.0%)
Age in months median (IQR)	7.3(5.6–9.0)	7.7(5.4–9.4)	7.4(5.5–9.1)
Travel time (minutes) to facility (median IQR)	39(16–63)	47(25–72)	41(18–65)
Peri-urban area of residence	1,189 (34.6%)	223 (22.1%)	1,412 (31.8%)
Use safe water	1331 (38.7%)	328 (32.6%)	1,659 (37.3%)
Treats drinking water	1959 (57.0%)	519 (51.5%)	2,478 (55.8%)
Hand-washing facility in a household	1174 (34.2%)	355 (35.3%)	1,529 (34.4%)
Ownership of latrine/toilet by a household	2143 (62.4%)	576 (57.2%)	2,719 (61.2%)
Has a birth certificate	159 (4.6%)	62 (6.2%)	221 (5.0%)
Is an orphan	67 (2.0%)	26 (2.6%)	93 (2.1%)
Sleep under mosquito-treated net	3247 (94.5%)	889 (88.3%)	4,136 (93.1%)
More than 1 h travel time	959 (27.9%)	375 (37.2%)	1,334 (30.1%)

### Pentavalent vaccination coverage

**Fig. 3:**
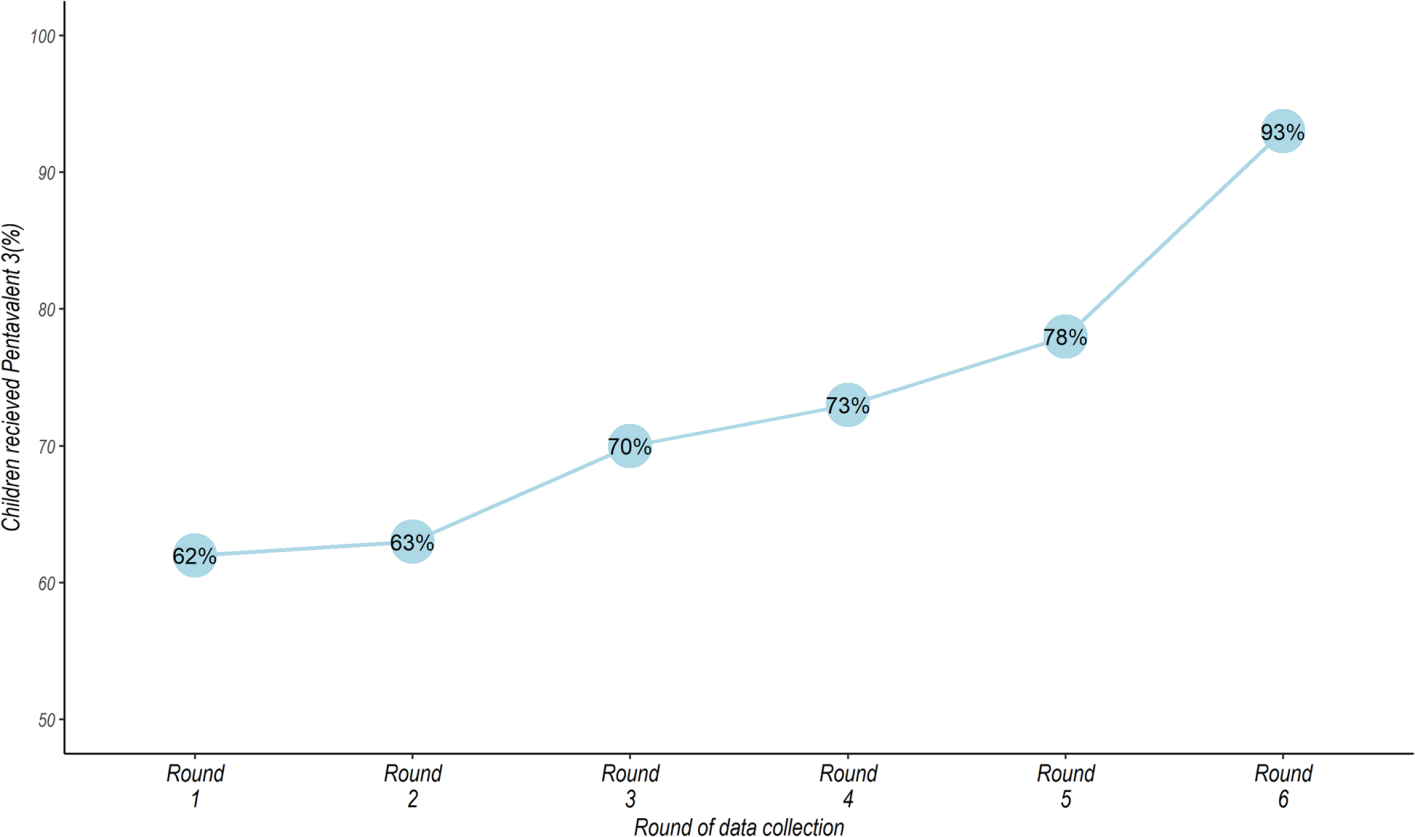
Vaccination coverage over the rounds of data collection in the community demographic surveillance system

**Fig. 4:**
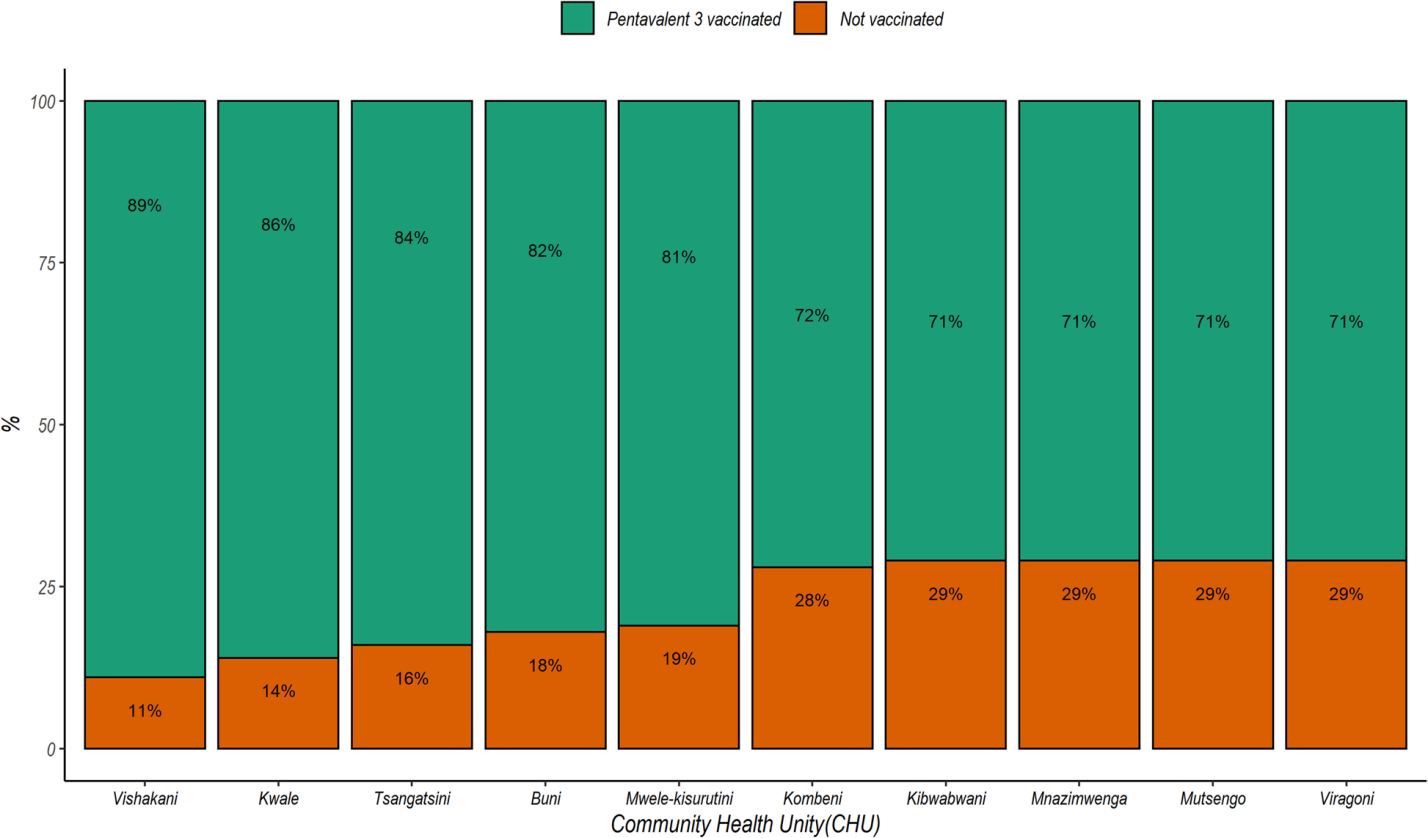
Vaccination coverage across community health units participating in the demographic surveillance system

We observed that coverage of pentavalent vaccination in the cohort improved over time (rounds of data collection) from 62% in January to June 2017 (round 1) to 93% in July to December 2019 (round 6) (see Fig. 3). The average coverage during the period was 3435/4442 (77.3%), and this varied across CHUs from 70.9% to 88.8% (see Fig. 4).

### Travel time to a health facility

**Fig. 5:**
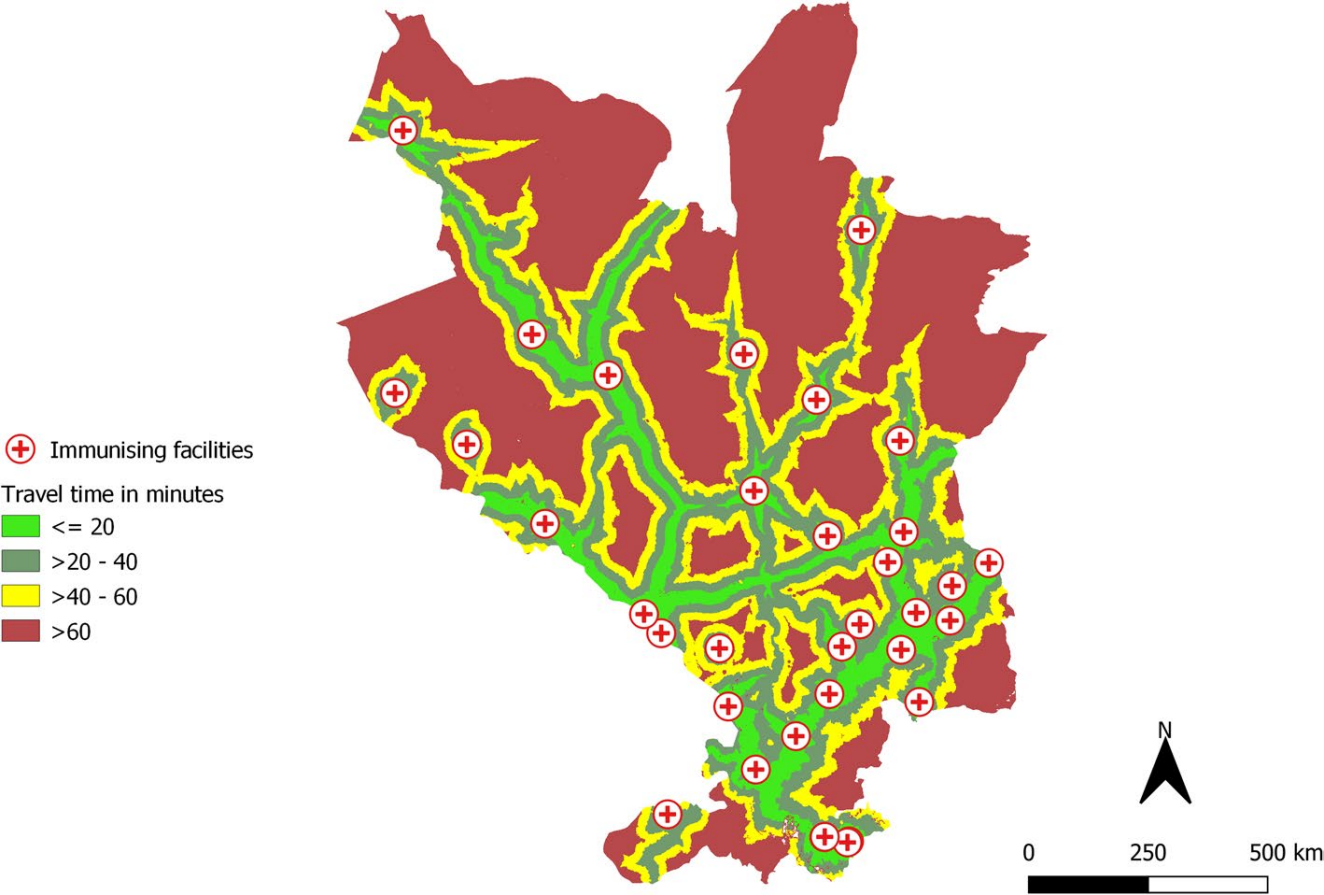
Distribution of the mean travel time from each grid (30 × 30 m) to the nearest immunizing health facility (red cross). The travel time was composite of walking and motorized transport to the nearest immunizing health facility in the study area. Source of map: *generated by the author using open-source software QGIS v3.12*

**Fig. 6:**
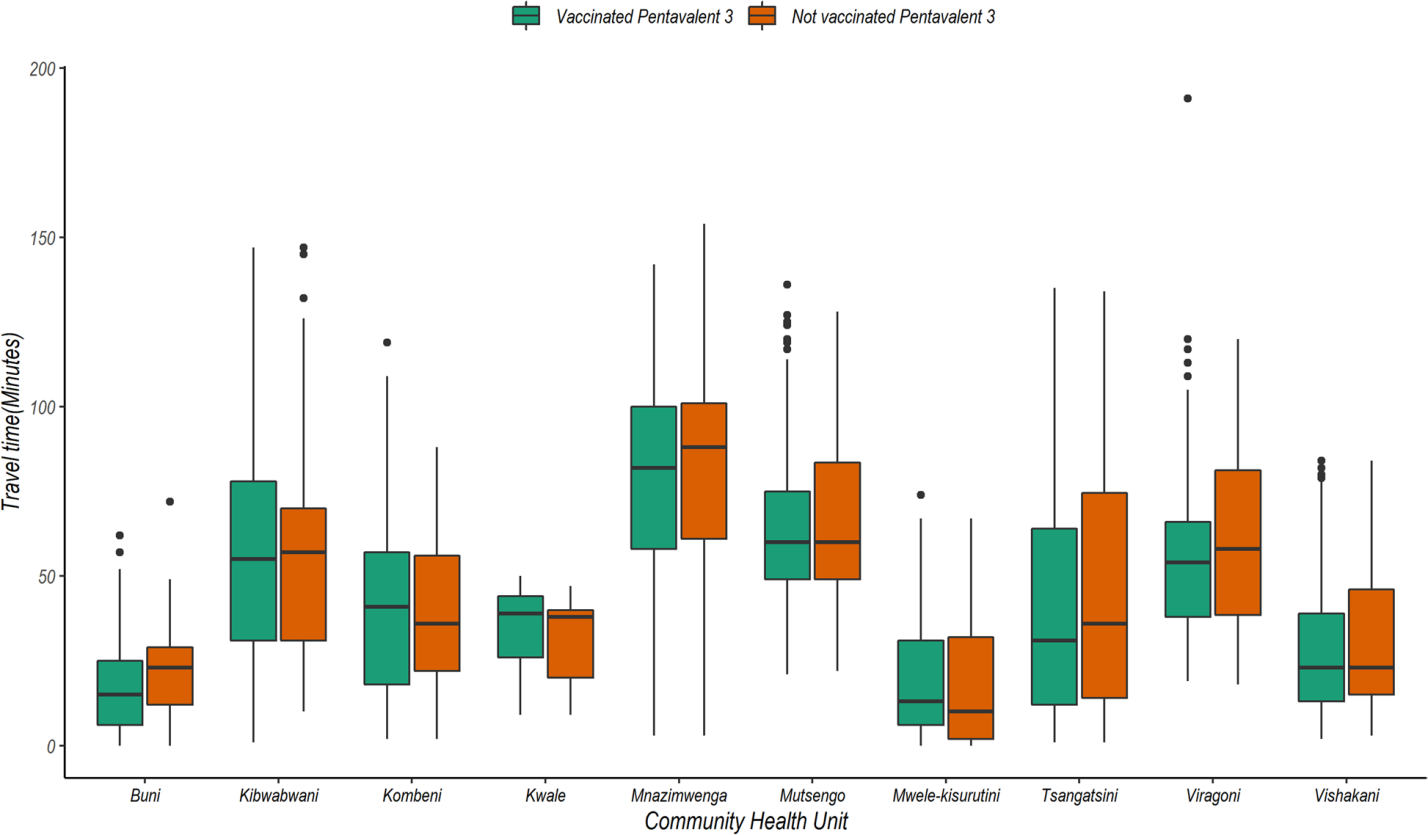
Travel time in different populations (vaccinated vs not vaccinated) across community health units participating in the demographic surveillance system

Within the study area, there were a total of 32 health facilities. Figure 5 provides a visual representation of the travel time to the nearest health facilities using the combined modes of transport (walking/cycling and motorized transport). The median travel time to a health facility was 41 (IQR = 18 – 65) minutes, and a total of 1266 (28.5%) children lived more than one-hour of travel-time to a health facility. Comparing the travel time across different CHUs, we observed households in CHUs bordering peri-urban areas namely Mwele, Buni and Vishakani had relatively lower travel time as compared to the CHUs in rural areas as shown in Fig. 6. Across all CHUs, we also observed that children who were vaccinated had relatively lower travel time as compared to non-vaccinated children.

### Factors influencing pentavalent vaccination coverage

Increased mean travel time to immunizing health facilities was associated with reduced odds of being vaccinated. Children who lived 30 min of travel from the health facilities had a pentavalent coverage of 82.6% compared to a coverage of 62.1% in children with longer travel times (more than 2 h from the health facility). A travel time of more than one hour to a health facility significantly reduced the likelihood of pentavalent vaccination by approximately 16% after adjusting for other factors (adjusted odds ratio = 0.84 (95% CI 0.74 – 0.94). In comparing travel time between type of residence, we observed that the median travel time was 54 (IQR 33–75) minutes in rural settlements and 17 min (IQR 8–31) in peri-urban settlements *(p* < *0.001)*. Other factors included in the model namely sleeping under treated mosquito net, vitamin A supplement, and ownership of birth certificates, were associated with an increased likelihood of pentavalent vaccination. Factors such as child sex and type of settlement (rural or peri-urban), were not significant predictors of pentavalent vaccination. The results from the multivariable model are shown in Table 2

**Table 2:** Multivariate model for factors influencing Pentavalent 3 vaccination coverage

**Characteristics**	**Adjusted odds ratio (95% CI)**
Urban area of residence (Urban)	1.02 (0.73 – 1.44)
Treats drinking water	1.09 (0.97 – 1.21)
Hand-washing facility in a household	0.82 (0.74 – 0.92) ^a^
Ownership of latrine/toilet by a household	1.04 (0.93 – 1.17)
Has a birth certificate	1.27 (1.00 – 1.61) ^a^
Is an orphan	0.80 (0.59 – 1.10)
Child sex (Male)	1.01 (0.93– 1.11)
Sleep under mosquito-treated net	1.36 (1.13 – 1.65) ^a^
Given vitamin A supplements	6.41 (5.82 – 7.07) ^a^
More than 1 h travel time	0.84 (0.75 – 0.94) ^a^

^a^denotes statistical significance at the 5% significance level

## Discussion

We sought to determine the pentavalent vaccination coverage, and estimate travel time to health facilities offering immunisation services, and explore its effect on immunisation coverage in one of the predominantly rural counties on the coast of Kenya using data from a community demographic and health surveillance system. The data from the surveillance system showed that slightly over three-quarters of the eligible children had received full pentavalent vaccination. While this immunisation coverage is commendable, it was below the GVAP goal of achieving 90% by year 2020 [8]. Recognizing that the pentavalent vaccine is primarily administered during routine immunization at health centres, we hypothesized that geographical accessibility was a key factor in determining pentavalent vaccination uptake. We observed that the mean travel time to a facility was 44.9 (SD = 31.2) minutes, assuming a composite mode of transport of walking/cycling and motorised transport. This varied significantly by place of residence (rural and peri-urban). We also noted that 28.5% of children lived more than one hour of travel from a health facility, which is far below the Kenyan policy recommendation that states that 90% of the population should live within one hour of walking speed from a health facility that offers immunisation services [39]. Travel times of more than one hour to a health facility were significantly associated with reduced odds of receiving pentavalent vaccination (AOR = 0.84 (95% CI 0.74 – 0.94), and travel times of more than two hours were associated with a Pentavalent coverage ratio of 62.1%, which is below the set target.

Previous studies on the barriers of accessing healthcare [40, 41] have shown that the time required to travel to a healthcare facility, particularly in sub-Saharan Africa, is the main barrier to accessing healthcare. As used in previous studies [11–13, 42, 43], we used a combination of walking, cycling and motorised transport to estimate the travel times to health facilities that offered immunisation services. The effect of spatial access on immunisation coverage has been explored by previous studies, and they have shown that travel time influences the uptake of child vaccination. In addition to geographical accessibility, a number of studies have also shown that child birth order, wealth quintiles, and exposure to media content positively influence immunisation coverage, especially in low- and middle-income countries [44–46], although our previous work in the area has shown that socioeconomic status is not associated. However, in this study, we did not explore these factors, as we were only interested in estimating the effects of geographical accessibility on immunisation coverage with a view of making recommendations to the local government to evenly increase and space out the number of health facilities that offer immunisation services in the area.

The involvement of community health workers/community health volunteers in childhood vaccination has been shown to be both efficient and cost-effective in expanding immunisation coverage and improving reporting systems, especially in hard-to-reach areas [47]. Our data demonstrated a marked improvement of pentavalent coverage over time since the inception of the community surveillance system implemented by CHVs, and whose data were used in this study (see Fig. 3). This further demonstrates the value addition to immunization coverage that CHVs’ involvement in child immunization services can offer. In this study, the use of CHVs, coupled with integrated audit and feedback activities embedded in the community by the CHVs could have improved the overall adoption of recommended immunization practices over time [48]. We posit that engaging CHVs in regular data collection in the households provided for increased contact with household members, which afforded them opportunities for enhanced health education and promotion, including tracing defaulters of essential health services such as vaccinations. We also noted marked differences in immunisation coverage in different CHUs, which could be due to group dynamics and subtle geographical differences within the study area [49]. Factors such ignorance of the need for immunisations, missing return dates for the next immunization schedule, fear of adverse events following immunisation, negative attitudes of health care providers and missed opportunities for vaccination have also been highlighted as factors that contribute to low vaccination coverage [50]. We found that other factors such as sleeping under treated mosquito net, vitamin A supplement and ownership of birth certificates, were associated with pentavalent vaccination. Even though these were included as potential confounders to the travel time – pentavalent vaccination relationship, they are nevertheless surrogates of positive health behaviours and as such markers for likelihood to take up health interventions, including childhood immunization.

### Study limitations

This study had several limitations. First, travel time estimations did not consider factors that might affect travel speed, especially in the rainy season, frequency of transport services, and traffic flow. The choice of confounding factors was also influenced by availability of surveillance data for this cohort and as such the explored relationship could partially be due to unmeasured confounding by other factors. We did not have data on birth order and access to media, which have been shown to affect vaccination coverage in other studies. However, our previous work in the area has shown that this community is generally poorer than the rest of the country (low social economic status in Principal Component Analyses, skewed towards poverty relative to the rest of the country) and as such assumed poorer access to print or electronic media [37]. For birth order, we assume that this was normally distributed given the large size of target population over several years and as such had adequate variability to not confound the explored relationship. Other nuances that are likely to affect care-seeking behaviour, such as variation of the quality of healthcare services [51], health professionals’ strikes [52], and stock-outs, could not be adjusted for in the model. To determine pentavalent coverage, we used data from the vaccination card and mother’s recall in the absence of the vaccination card. The inclusion of maternal recall potentially introduced recall bias.

## Conclusion and recommendation

We found that pentavalent coverage was at 77%. The median travel time to a health facility was 41 min, and about a third of the children lived more than one-hour travel-time to a health facility. Coverage was significantly affected by geographical access to health facilities that offer immunization services with travel times of more than one hour to a health facility significantly associated with reduced odds of receiving pentavalent vaccine.

To improve immunisation coverage, especially for pentavalent, a high-resolution map of estimated travel time to the nearest healthcare facility could be used by local health authorities, policy makers and relevant stakeholders to identify potential locations for immunization centres improve physical accessibility in this community. Other interventions could include improving the road network and/or stepping up health outreach activities that include vaccinations in hard-to-reach areas within the county.

## Data Availability

Data are available upon a reasonable request to the institutional data governance committee.

## Abbreviations

BCG: Bacille Calmette -Guerin
CHU: Community Health Unit
CHV: Community Health Volunteer
EPI: Expanded Programme of Immunization
GAVI: Global Alliance for Vaccines and Immunizations
GVAP: Global Vaccine Action Plan
GPS: Global Positioning System
GMM: Google Map Maker
ODK: Open Data KitOSM – OpenStreetMaps
SSA: Sub-Saharan Africa
WHO: World Health Organization DTP: Diphtheria-Tetanus-Pertussis
